# The role of microRNAs in human neural stem cells, neuronal differentiation and subtype specification

**DOI:** 10.1007/s00441-014-1981-y

**Published:** 2014-08-30

**Authors:** Laura Stappert, Beate Roese-Koerner, Oliver Brüstle

**Affiliations:** Institute of Reconstructive Neurobiology LIFE & BRAIN Center, University of Bonn and Hertie Foundation, Sigmund-Freud-Straße 25, Bonn, 53127 Germany

**Keywords:** microRNAs, Neural development, Cell fate specification, Human neural stem cells, Neuronal subtypes

## Abstract

The impressive neuronal diversity found within the nervous system emerges from a limited pool of neural progenitor cells that proceed through different gene expression programs to acquire distinct cell fates. Here, we review recent evidence indicating that microRNAs (miRNAs) are critically involved in conferring neural cell identities during neural induction, neuronal differentiation and subtype specification. Several studies have shown that miRNAs act in concert with other gene regulatory factors and genetic switches to regulate the spatial and temporal expression profiles of important cell fate determinants. So far, most studies addressing the role of miRNAs during neurogenesis were conducted using animal models. With the advent of human pluripotent stem cells and the possibility to differentiate these into neural stem cells, we now have the opportunity to study miRNAs in a human context. More insight into the impact of miRNA-based regulation during neural fate choice could in the end be exploited to develop new strategies for the generation of distinct human neuronal cell types.

## Introduction

Initially considered as “junk RNA”, non-coding RNAs are currently perceived as critical regulators of the cellular homeostasis (reviewed by Esteller 2011). In particular, microRNAs (miRNAs), which constitute a distinct class of small non-coding RNAs, have emerged as important post-transcriptional gene regulators. Mature miRNAs arise from large primary transcripts containing hairpin structures that are further processed by the sequential action of two ribonuclease (III) enzymes: Drosha and Dicer. The mature miRNAs are then incorporated into the RNA-induced silencing complex (RISC) and serve as guides to target mRNAs for translational inhibition or mRNA degradation. To date, more than 2,500 miRNAs have been annotated for the human genome (miRBase annotation v20; Kozomara and Griffiths-Jones 2011, 2013) and a large fraction of the known miRNAs is expressed in the human brain (Shao et al. 2010). Considering that each of these miRNAs is predicted to recognize several hundreds of targets, a large proportion of the transcriptome and consequently many cellular processes might be subjected to miRNA-based regulation (Lewis et al. 2005; reviewed by Esteller 2011). This is also the case for the mediation of cell fate decisions, where miRNAs act in synergy with other transcription regulators (transcription factors and epigenetic regulators) to establish gene regulatory networks (Herranz and Cohen 2010; Peláez and Carthew 2012; Arora et al. 2013). In this context, miRNAs and transcription factors can form feed-back or feed-forward loops. Feed-back regulation can be either negative (e.g., a transcription factor *limits* its own expression by inducing the expression of its own negative miRNA regulator) or positive (e.g., a miRNA *reinforces* its own expression by targeting its own negative transcription factor regulator). Double-negative feed-back loops, in which the miRNA and the transcription factor reciprocally repress each other, can function as bi-stable switches. Neuronal subtype decisions, in particular, often depend on pairs of cross-repressive transcription factors that might be regulated by miRNAs (e.g., Chen et al. 2011). Feed-forward loops are more complex and consist in two paths of regulation—one direct and one indirect—that can either act in the same (coherent) or in opposite directions (incoherent). MicroRNAs may be also components of feed-forward loops, whereby several different combinations are possible (for a detailed description see Peláez and Carthew 2012). On the one hand, miRNAs may help to ensure the robustness of a gene regulatory network by dampening perturbations and reducing noise. For instance, it was recently shown that miR-9 reduces the impact of genomic variations in *Drosophila* (Cassidy et al. 2013). On the other hand, miRNAs may also function as critical switches to canalize gene expression during cell fate decisions. This has been nicely demonstrated by the role of miRNAs in establishing chemosensory neuron asymmetry in *C. elegans* (reviewed by Alqadah et al. 2013).

In this review, we will discuss how miRNAs interact with gene regulatory motifs to regulate neuronal fate decisions. In the first part, we focus on the impact of miRNAs during neural induction and exemplarily highlight the interaction of miR-124 and miR-9 with important regulatory circuits and epigenetic regulators. In the second part, we describe how miRNAs interact with spatial and temporal fate determinants to generate the neuronal diversity found in the central nervous system (CNS). Finally, we will discuss how this knowledge could be harnessed to employ miRNA-based regulation for the derivation of specific neuronal subtypes from human pluripotent stem cells or neural stem cells.

## MicroRNAs interact with gene regulatory motifs to regulate neural induction and neuronal differentiation

Many of the miRNAs expressed in the CNS are dynamically regulated both during physiological brain development and in vitro neural differentiation of stem cells, indicating a significant contribution to neural development and function (Krichevsky et al. 2003, 2006; Sempere et al. 2004; Miska et al. 2004; Smith et al. 2010; Liu et al. 2012). Indeed, the emerging picture is that miRNAs play critical roles throughout neural development from neural induction to neural progenitor expansion, differentiation and neuronal subtype specification (reviewed by Sun et al. 2013; Bian et al. 2013). Furthermore, miRNAs are also involved in regulating neuronal migration (e.g., Gaughwin et al. 2011; Rago et al. 2014) as well as neuronal function, neurite outgrowth and synaptic plasticity (reviewed by Siegel et al. 2011; McNeill and Van Vactor 2012). The overall impact of miRNAs as essential regulators of differentiation and neural development was first demonstrated by global loss-of-function experiments via deleting key components of the miRNA processing machinery, i.e., Dicer or Drosha co-factor DGCR8 (Kanellopoulou et al. 2005; Giraldez et al. 2005; Wang et al. 2007; Davis et al. 2008). Since then, several laboratories have taken advantage of the newly developed techniques to selectively modulate the activity of specific miRNAs in order to dissect their functions (reviewed by Akerblom et al. 2012). Nevertheless, considering the large numbers of miRNA species expressed in the CNS, knowledge on miRNA-based regulation during neurogenesis is still at its dawn. This is even more true for human neural development, which, until recently, was not accessible to standardized in vitro experimentation. With the increasing availability of human neural cell types from human pluripotent stem (hPS) cells, there is now the opportunity to study miRNAs in association with human physiology (reviewed by Benchoua and Peschanski 2013). A deeper insight into the role of miRNAs during human neural fate determination could, in the end, also be exploited to develop refined protocols for the generation of specific human neural subtypes.

### MicroRNAs regulating the transition of pluripotent stem cells to the neural lineage

When induced to enter neural differentiation, hPS cells undergo specific fate transitions reminiscent of in vivo neural development. This includes the transition of hPS cells to neuroepithelial cells, their segregation into distinct neural progenitors and terminal differentiation into specific neuronal and glial cell types. Furthermore, hPS cells respond to the same extracellular cues regulating neural development in vivo. For instance, during development, neural induction relies on the inhibition of the Activin/TGFβ-mediated pluripotency pathways and the anti-neural effects of BMP (reviewed by Stern 2005). Accordingly, pharmacological blockage of BMP/TGFβ signaling can be used to strongly promote the conversion of hPS cells towards the neural lineage (e.g., Lee et al. 2007; Smith et al. 2008; Chambers et al. 2009). This approach has been designated “dual SMAD inhibition”, since both BMP and Activin/TGFβ signaling converge on SMAD proteins as main signal transduction molecules (Chambers et al. 2009).

**Fig. 1:**
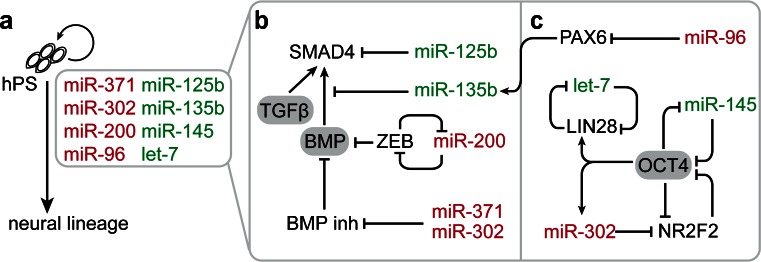
Schematic representation of miRNA-target interactions regulating neural lineage entry of hPS cells. (**a**) Overview of the miRNAs contributing to neural induction by influencing the activity of anti-neural BMP/TGFβ signaling (**b**) or by directly regulating the expression of pluripotency- and neural fate-associated transcription factors (**c**; miRNAs labeled in *red* have an inhibitory and miRNAs in *green* a promoting effect on neural induction. (**b**) Both miR-302 and miR-371 potentiate BMP signaling via targeting BMP inhibitors, thus creating a barrier for neural induction. Likewise, miR-200 promotes BMP signaling as part of a double-negative feed-back loop with the BMP repressor ZEB. In contrast, miR-125b and miR-135b interfere with BMP/TGFβ signaling by targeting SMAD4 and other important components of the BMP/TGFβ signaling cascade leading to an enhanced neural lineage entry. (**c**) In addition to its impact on BMP signaling, miR-302 also acts in concert with OCT4 to ensure repression of pro-neural NR2F2. Reciprocally, NR2F2 represses OCT4 expression, forming a double-negative feed-back loop. OCT4 directly represses miR-145 expression and indirectly inhibits let-7 maturation via induction of Lin28 expression. In turn, both miR-145 and let-7 repress the expression of pluripotency factors and promote differentiation. In contrast, miR-96 interferes with neural induction by targeting the neural lineage determinant PAX6. PAX6, in turn, activates other neuronal transcription factors and miR-135

Several miRNAs, which target components or modulators of the BMP/TGFβ signaling cascade, have been identified to either positively or negatively affect the neural lineage entry of hPS cells (reviewed by Benchoua and Peschanski 2013) (Fig. 1a, b). On the one hand, neural induction is promoted by miR-125a/b and miR-135b, which target key components of the BMP/TGFβ signaling cascade including different receptors and SMAD signal transduction molecules (Boissart et al. 2012; Bhinge et al. 2014). On the other hand, miR-302/367 blocks neural induction and contributes to a higher ground-state level of BMP signaling, by targeting several endogenous inhibitors of the pathway, such as *Lefty, DAZAP2, SLAIN1* and *TOB2* (Rosa et al. 2009; Lipchina et al. 2011). Similarly, miR-371 may indirectly increase BMP activity in hPS cells via targeting BMP repressors (Kim et al. 2011). In fact, certain hPS cell lines are characterized by elevated levels of miR-371, which is accompanied by a higher resistance to neural induction (Kim et al. 2011). MiR-200 acts on the same pathway and represses neural induction of hES cells by targeting the transcription factor *ZEB*–a negative regulator of BMP/TGFβ signaling (Du et al. 2013). In turn, expression of the miR-200 family is inhibited by ZEB transcription factors forming a double-negative feed-back loop (Burk et al. 2008). MicroRNAs may also directly modulate expression of transcription factors essential for either neuroectoderm specification or pluripotency (Fig. 1c). For instance, miR-96 specifically inhibits neural induction of hES cells by targeting the transcription factor *PAX6* (Du et al. 2013). PAX6, in turn, activates the expression of other neural fate-associated transcription factors as well as of miR-135b, which was recently shown to contribute to neural lineage entry (Bhinge et al. 2014). Another example is miR-302/367, which, besides its role in de-repressing the BMP pathway, represses the pro-neural transcription factor *NR2F2* (Rosa and Brivanlou 2011). In this context, miR-302 may act as a second layer of regulation next to OCT4, which induces miR-302 expression but also directly represses *NR2F2* transcription. In turn, NR2F2 represses *OCT4* transcription during differentiation and thus reinforces its own expression. MiR-145, instead, promotes the differentiation of hES cells into mesodermal and neuroectodermal lineages as part of a double-negative feed-back loop with OCT4 (Xu et al. 2009). In undifferentiated hES cells, expression of miR-145 is repressed by OCT4. Upon differentiation, miR-145 is up-regulated leading to the down-regulation of *OCT4* and other pluripotency genes by direct targeting (Xu et al. 2009). Another potent inhibitor of pluripotency and promoter of the neural lineage is the let-7 miRNA family (reviewed by Greve et al. 2013 and Rehfeld et al. in this Special Issue). In ES cells, processing of let-7 intermediates and thus mature let-7 expression is compromised due to the action of Lin28A and Lin28B (Rybak et al. 2008; Heo et al. 2009; Piskounova et al. 2011). In neural progenitor cells, expression of *Lin28* is down-regulated allowing mature let-7 to accumulate, which is reinforced by let-7 targeting its own negative regulator *Lin28* (Guo et al. 2006; Rybak et al. 2008). For further insights into the impact of the Lin28-let-7 bistable switch during neural induction and differentiation, please refer to the review by Rehfeld et al. in this Special Issue.

### MicroRNAs regulating the balance between neural progenitor self-renewal and differentiation

**Fig. 2:**
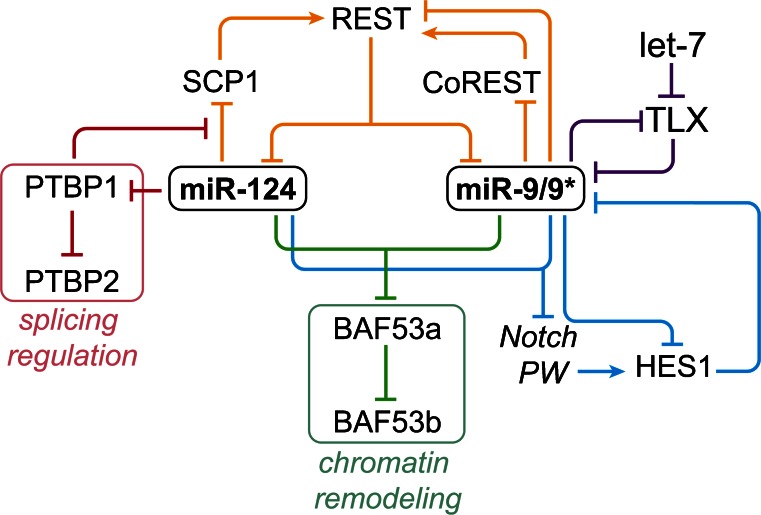
MiR-124 and miR-9/9* engage in complex regulatory circuits activating a neuronal gene expression program. Expression of miR-124 and miR-9/9* is controlled by the neurogenic repressor REST and its co-factors SCP1 and CoREST. In addition, miR-9/9* is repressed by TLX and the Notch effector HES1. During neuronal differentiation, miR-124 and miR-9/9* are up-regulated and reinforce their own expression by targeting their negative regulators. For instance, miR-9 forms auto-regulatory loops with HES1 and the let-7 target TLX. Both miR-124 and miR-9/9* repress the expression of additional components of the Notch pathway (PW). Furthermore, forced expression of miR-124 and miR-9/9* induces a switch of epigenetic regulators. MiR-124 and miR-9* favor the switch from BAF53a to BAF53b to be included in the BAF chromatin remodeling complex leading to the induction of dendritic outgrowth. In addition, miR-124 targets the mRNA splicing regulator PTBP1 allowing the expression of the neuron-enriched homolog PTBP2, which induces a neuron-specific pre-mRNA splicing pattern. Down-regulation of PTBP1 also leads to the abolishment of its inhibitory impact on the interaction of miR-124 with REST co-factor SCP1

Once the neural fate is induced, a highly orchestrated network of developmental cues regulates the proliferation, differentiation and spatial distribution of neuronal progenitors. The abundance of these players is fine-tuned by a certain set of brain-enriched miRNAs. While miR-124, miR-125b, miR-137, miR-9 and let-7 have been shown to promote neuronal differentiation, other miRNAs, such as miR-134 and miR-184, have been implicated in neural progenitor maintenance and proliferation (for a detailed review see, e.g., Bian et al. 2013). Furthermore, miRNAs may regulate the shift from neuronal to glial fate and promote the generation of astrocytes or oligodendrocytes (for review see, e.g., He et al. 2012b; Zheng et al. 2012). Among the brain-enriched miRNAs, the functions of miR-124 and miR-9 in promoting neuronal differentiation have been extensively studied (reviewed by Coolen et al. 2013; Akerblom and Jakobsson 2013; see also Abernathy and Yoo, this Special Issue). Both miRNAs interact with gene regulatory networks and genetic switches to induce the expression of a neuronal differentiation program (Fig. 2). As already shown in 2005, overexpression of miR-124 in Hela cells is able to alter their expression profile to resemble that of neuronal cells (Lim et al. 2005). About 5 years later, it was demonstrated that it is possible to actually transdifferentiate fibroblasts and other somatic cells into so-called induced neurons by overexpression of specific neurogenic transcription factors (Vierbuchen et al. 2010; Pang et al. 2011). Interestingly, this direct neuronal conversion process can be further supported by miR-124 or even induced by solely overexpressing miR-124 and miR-9/9*, indicating that these miRNAs can be instructive for the neuronal fate (Ambasudhan et al. 2011; Yoo et al. 2011). The function of miR-124 and miR-9/9* during neuronal conversion may–at least in part—be based on their cooperative influence on the ATP-dependent BAF chromatin remodeling complex. The subunit composition of this complex differs between neural progenitors and post-mitotic neurons. One of the main components exchanged upon neuronal differentiation is BAF53a, which is replaced by its homolog BAF53b. Ectopic expression of miR-9* and miR-124 induces the down-regulation of *BAF53a* allowing the incorporation of BAF53b, which is also essential for dendrite outgrowth (Yoo et al. 2009). A similar miRNA-mediated switch was shown for *PTBP1*, which is expressed in neural progenitors and its homolog *PTBP2*, which is expressed in neurons. The RNA binding Polypyrimidine tract-binding proteins (PTBPs) impact on mRNA transcription, localization, stability and modification. They were even found to affect miRNA activity by altering the secondary mRNA structure and competing with the binding to miRNA target sites (Xue et al. 2013). PTBP1 interferes with *PTBP2* expression at the level of *PTBP2* mRNA splicing. MiR-124-induced repression of *PTBP1* releases this inhibitory effect allowing the expression of *PTBP2*, which in turn promotes the switch to a neuron-specific alternative splicing program (Visvanathan et al. 2007). Depletion of the PTBP1 activity in non-neuronal cells was found to be sufficient to also initiate neuronal conversion (Xue et al. 2013). This might be partially attributed to the fact that PTBP1 blocks miRNA-mediated regulation of another repressor of neurogenesis–the repressor-element-1 silencing transcription factor (REST) complex (Xue et al. 2013). PTBP1 competes with miR-124 and miR-96 for binding to the mRNA of REST co-factor *SCP1* (Xue et al. 2013). Conditional knock-out of *REST* in fibroblasts is sufficient to induce elevated expression of neural genes but does not induce a shift in cellular identity as fibroblast-specific genes are still expressed (Aoki et al. 2012). During neurogenesis, *REST* itself underlies post-transcriptional regulation via a functional binding site for miR-9 in its 3’UTR (Packer et al. 2008). In addition, miR-9*–a functional miRNA produced from the same hairpin precursor as miR-9–regulates *CoREST*, yet another essential REST co-factor. In turn, REST represses the expression of neuronal genes and neuronal miRNAs including miR-124 and miR-9/9* (Wu and Xie 2006; Conaco et al. 2006; Otto et al. 2007). Taken together, a picture emerges in which miR-124 and miR-9/9* are in the center of a complex regulatory circuit involving the BAF53a/BAF53b and PTBP1/PTBP2 switch motifs, and a double-negative feed-back loop with REST (Fig. 2).

The regulation of neural progenitor proliferation by miR-9 is based on a whole network of other interaction partners. Another node in this complex interplay is a feed-back loop with the orphan nuclear receptor *TLX/NR2E1* (Fig. 2). This transcription factor regulates the maintenance and self-renewal of adult neural stem cells via recruitment of HDAC repressors to the promoters of target genes like *p21* and *Pten* (Sun et al. 2007). TLX has been shown to repress miR-9 expression, while miR-9 reduces TLX protein levels in adult neural stem cells (Zhao et al. 2009). However, data from miR-9_2/miR-9_3 knock-out mice suggest that, depending on the developmental stage, miR-9 can associate with the RNA-binding protein Elavl1 to enhance *TLX* translation instead (Shibata et al. 2011). It has been shown that let-7b and let-7 days target *TLX* as well and thus might be able to trigger differentiation by de-repression of miR-9 (Zhao et al. 2010, 2013). In addition to its association with Elavl1, miR-9 competes with Elavl2 for binding to target mRNAs. Elavl2 binds to U-rich regions of *FoxG1* mRNA, thereby dampening miR-9-mediated *FoxG1* suppression (Shibata et al. 2011).

Notch signaling is one of the key pathways regulating neuronal development and expansion of neural progenitors. Both miR-124 and miR-9 have been shown to target several components of the Notch signaling cascade (Fig. 2). While miR-124 targets the Notch ligand *Jag1* (Liu et al. 2011) and the Notch down-stream effector *Sox9* (Cheng et al. 2009), miR-9 regulates Hes gene family members (Leucht et al. 2008; Bonev et al. 2011, 2012; Coolen et al. 2012). In turn, levels of miR-9 seem to depend on Notch signaling, building up yet another feed-back loop (Coolen et al. 2012; Bonev et al. 2012). In murine neural progenitors, this feedback induces an out-of-phase oscillation of pri-miR-9 and Hes1 levels (Bonev et al. 2012). However, mature miR-9 accumulates over time, limiting the oscillatory period. Presumably, this mechanism participates in timing the differentiation of neural progenitors, since neuronal differentiation is accompanied by high levels of miR-9 and low *Hes1* levels (Bonev et al. 2011, 2012). Interaction of miR-9 with the Notch signaling pathway has also been found in *Drosophila*. Here, *Drosophila* homolog miR-9a impacts on Notch-mediated lateral inhibition during specification of sensory organ precursors by targeting of *dLMO* (Li et al. 2006; Biryukova et al. 2009) and *senseless* (Cassidy et al. 2013). However, these data have to be interpreted carefully, as, in *Drosophila* miR-9, expression is restricted to epithelial cells (Li et al. 2006) and, therefore, does not reflect the brain-enriched expression profile found in vertebrates (Sempere et al. 2004; Miska et al. 2004; Wienholds et al. 2005).

The data gathered so far illustrate that miR-124 and miR-9/9* add an additional level of intricacy to the highly orchestrated networks underlying neuronal differentiation. However, the list of miR-9 and miR-124 targets is still growing (for a detailed description of additional genes regulated by miR-9/9* and miR-124, please refer to the reviews by Coolen et al. 2013; Akerblom and Jakobsson 2013).

## The role of microRNAs during neuronal subtype specification

Besides their general impact on neuronal differentiation, miRNAs contribute to the neuronal diversity found in the CNS. The vertebrate brain consists in many different neuronal subtypes with distinct neurotransmitter phenotypes, functions and innervation targets. These diverse subtypes develop from an initially rather limited variety of multipotent neural progenitor cells. During neural development, neural progenitor cells adopt different spatial identities along the antero-posterior (AP) and dorso-ventral (DV) axis of the neural tube and subsequently generate distinct neuronal and glial subtypes. Depending on their position within the AP and DV coordinates, neural stem cells are exposed to specific morphogens, such as SHH, FGFs, Wnts and BMPs, which are secreted by organizing centers (reviewed by Le Dréau and Martí 2012). Based on the combination of these signal gradients and intrinsic cues, neural stem cells activate specific transcriptional programs determining their competence, i.e., their range of neural subtype progenies (reviewed by Fishell and Heintz 2013; Kohwi and Doe 2013). Furthermore, neural progenitors may acquire distinct temporal identities and may change their differentiation competence over time, as shown, for instance, during retinal and cerebral cortex development (reviewed by Kohwi and Doe 2013). The identity of the different neuronal cell types is determined by the combinatorial expression of transcription factors and modulated by other gene expression regulators, including miRNAs. Thereby, miRNAs might control genetic switches and regulate the expression of important cell fate determinants in a spatial and temporal manner. Furthermore, miRNAs might modulate the signaling dimensions of morphogens by targeting important components of their respective signaling cascades (reviewed by Inui et al. 2010, 2012). For an overview on miRNAs contributing to neuronal subtype decisions, see Table 1.

**Table 1:** MicroRNA–target interactions involved in neuronal subtype specification in vivo

	miRNA	Target	Function	Species	Reference
ASE chemo-sensory neurons	lys-6	COG-1	Induction of ASEL identity	*C. elegans*	Johnston and Hobert 2003; Johnston et al. 2005
	miR-273	DIE-1	Induction of ASER identity		
Mushroom body neurons	let-7, miR-125	chinmo, abrupt	Temporal control of neuronal subtype specification	*Drosophila*	Wu et al. 2012; Kucherenko et al. 2012
Anterior–posterior axis	miR-9	Hes1 (homologs)	Promotion of cell cycle exit and differentiation, specific impact on survival of forebrain but not hindbrain neuronal progenitors	*Xenopus*; zebrafish; mouse	Bonev et al. 2011, 2012; Coolen et al. 2012
Cortex	miR-9	FoxG1, several other targets	Important for the generation of Cajal Retzius cells and proper cortical layer formation	Mouse	Shibata et al. 2008, 2011
	^a^		MiRNA activity is necessary for proper generation of cortical layers	Mouse	Saurat et al. 2013
Retina	miR-129, miR-155, miR-214, miR-222	Xotx2, Xvsx1	Developmental timing of subtype specification	*Xenopus*	Decembrini et al. 2009
	let-7, miR-125, miR-9	Ptrg, Lin28b	Acceleration of progenitor fate progression towards late-born neurons	Mouse	La Torre et al. 2013
Olfactory bulb	miR-7a	Pax6	Restriction of DA neuron differentiation	Mouse	de Chevigny et al. 2012
Midbrain	miR-135a	Lmx1b	Delimiting the DV extent of the dopaminergic progenitor pool	Mouse	Anderegg et al. 2013
Midbrain–hindbrain boundary	miR-9	Fgfr1, Canopy, Fgf8, Her5, Her9	Maintenance and correct positioning of the midbrain-hindbrain boundary	Zebrafish	Leucht et al. 2008
Spinal cord	miR-17-3p	Olig2	Specification of the p2-pMN progenitor boundary	Mouse	Chen et al. 2011
	miR-196	Hoxb8	Spatial restriction of lumbar motor neuron identity	Chicken	Asli and Kessel 2010
	miR-9	FoxP1	Specification of spatial MN identity (LMC and MMC column)	Chicken	Otaegi et al. 2011; Otaegi et al. 2012
		OC1	Specification of temporal MN identity (switch from early- LMCm to late-born LMCI)	Chicken	Luxenhofer et al. 2014

*DA* dopaminergic, *DV* dorso-ventral, LMC lateral motor neuron column, *LMCl* lateral LMC subcolumn, *LMCm* medial LMC subcolumn, *MMC* medial motor neuron column, *MN* motor neuron

^a^ Inferred from Dicer knock-out experiments, no specific miRNA identified so far

The first evidence pointing to a modulatory role of miRNAs in programming neuronal identities came from studies in *C. elegans* (Johnston and Hobert 2003; Johnston et al. 2005). There are two classes of *C. elegans*, ASE chemosensory neurons, which are located at the right (ASER) or left (ASEL) side of the worm’s head. Although these neurons share many characteristics with regard to their projection and gene expression profiles, they are functionally divergent and react to different environmental cues. This left–right asymmetry is established by a pair of miRNAs (lys-6, miR-273) and their transcription factor targets (*DIE-1*, *COG-1*), which together form a cross-repressive loop. ASEL neurons show high expression levels of lys-6, which directly represses the ASER-promoting transcription factor *COG-1*. Low COG-1 expression levels allow for the expression of DIE-1 transcription factor, which induces the expression of ASEL genes including lys-6, while repressing the expression of ASER-associated genes. In turn, ASER neurons do not express lys-6 but high levels of *COG-1*, which induces the expression of miR-273. This miRNA targets *DIE-1*, thus leading to a de-repression of ASER genes (Hobert 2004; reviewed by Alqadah et al. 2013).

In the vertebrate CNS, some miRNAs exhibit region-specific expression patterns indicating that the different neuronal subtypes residing in these regions may express distinct miRNA profiles (Kapsimali et al. 2007; Landgraf et al. 2007; Kim et al. 2007). Recently, He et al. (2012a) succeeded in analyzing the active miRNA repertoire at a neuron subtype-specific resolution in the adult mouse brain. For this purpose, they used miRNA-tagging and affinity-purification (miRAP), which relies on Cre-induced cell-specific tagging of Argonaute 2 (AGO2) and subsequent co-immunopurification of the tagged-AGO2 and its associated miRNAs (He et al. 2012a). Using this approach, they could demonstrate substantial differences between the expressed miRNA repertoire of glutamatergic neurons and GABAergic interneurons co-expressing either parvalbumin (PV) or somatostatin (SST). For instance, miR-133b and miR-187 were found to be higher expressed in GABAergic neurons than in glutamatergic pyramidal neurons, whereby miR-133b was enriched in the PV-expressing and miR-187 in the SST-expressing GABAergic neurons.

Several studies have addressed the impact of global miRNA loss on the development of specific brain regions by knocking-out Dicer, the key enzyme of miRNA biogenesis. Using the Cre/loxP recombination system, different mouse models for region- or cell-type-specific depletion of Dicer have been developed, e.g., for the retina (Georgi and Reh 2010; for an overview, see Cremisi 2013), cerebral cortex (De Pietri et al. 2008; Saurat et al. 2013; Cremisi 2013), hippocampus (Li et al. 2011a), midbrain (Kim et al. 2007; Huang et al. 2010; Pang et al. 2014) and spinal cord (Zheng et al. 2010; Chen and Wichterle 2012). Interestingly, the impact of Dicer knock-out was variable with regard to the different brain regions targeted and the neuronal subtypes affected. For instance, conditional knock-out of Dicer during late-stage dopaminergic differentiation of mouse ES cells led to a complete loss of dopaminergic neurons, while the number of GABAergic neurons was only reduced by 50 % (Kim et al. 2007). The same study further demonstrated that Dicer depletion in mouse postmitotic midbrain dopaminergic neurons using a DAT-Cre line results in the progressive loss of these cells, which is accompanied by the development of Parkinson’s disease-like symptoms (Kim et al. 2007). A similar loss of dopaminergic neurons due to increased apoptosis was also observed upon specific Dicer deletion in the midbrain of postnatal mice using adenovirus/AAV2-mediated Cre-delivery (Pang et al. 2014).

While Dicer ablation studies have revealed the overall importance of miRNAs in the development and maintenance of different neuronal cell types, further studies have led to the identification of specific miRNAs involved in neuronal subtype specification (Table 1). In the following paragraphs, we will delineate how miRNAs contribute to specifying neuronal subtypes by regulating the spatial or temporal identity of neural progenitor cells. We will further focus on the impact of miRNA-based regulation on neuronal subtype specification in the spinal cord. Finally, we will discuss how miRNAs could be used as tools to modulate the generation of distinct neuronal cell types directly from hPS cells or from hPS cell-derived neural stem cells.

### MicroRNAs regulating the spatial identity of neural progenitors

The refinement of the CNS into its main subdivisions along the AP axis (forebrain, midbrain, hindbrain and spinal cord) is regulated by local organizing centers (Lumsden and Krumlauf 1996; Kiecker and Lumsden 2012). One of them, the midbrain–hindbrain boundary (MHB), also called isthmus, regulates the patterning of the midbrain and the anterior hindbrain via Wnt and FGF signaling (Wurst and Bally-Cuif 2001). In *Zebrafish*, levels of MHB effectors like *fgfr1*, *fgf8* and *canopy* as well as genes preventing MHB neurogenesis, i.e., *her5* and *her9*, are regulated by miR-9 (Leucht et al. 2008). Interestingly, the MHB is the only part of the *Zebrafish* neural tube where miR-9 cannot be detected (Leucht et al. 2008). Gain- and loss-of-function studies underlined the importance of miR-9 activity for the maintenance and correct positioning of this organizing center. While ectopic miR-9 expression delimits the spatial extent of the MHB, inhibition of miR-9 causes its expansion along the AP axis (Leucht et al. 2008). Protection of *fgfr1* from miR-9 targeting was sufficient to partially rescue MHB formation, underlining the importance of the FGF signaling pathway during this process. However, the effect of miR-9 overexpression on MHB marker expression occurred earlier and was even more pronounced than the changes observed in the *Zebrafish fgf8-*mutant *ace* indicating that more targets are at play (Leucht et al. 2008). Accordingly, target protection of the Hes homolog *Her5* alone in the presence of miR-9 was also able to rescue the expression of MHB markers (Leucht et al. 2008).

Functional targeting of Hes genes by miR-9 was shown to be conserved in *Xenopus* and mouse (Bonev et al. 2011, 2012; Coolen et al. 2012). In both organisms, miR-9 acts as a fine-tuner of neurogenesis as part of a negative feed-back loop with Hes genes as described in the first part of this review (Coolen et al. 2012; Bonev et al. 2012). In *Xenopus*, miR-9 loss causes a failure in neurogenesis along the AP axis by de-repression of the Hes1 homolog *hairy1* (Bonev et al. 2011, 2012). The resulting elevated levels of hairy1 were found to promote proliferation through Fgf8, Zic1 and CyclinD1 (Bonev et al. 2011). However, besides its general role in cell cycle exit, the impact of miR-9 on *Xenopus* neuronal progenitors differed, dependent on the region analyzed (Bonev et al. 2011, 2012). In the *Xenopus* hindbrain, miR-9 expression is restricted to neural progenitors, which expand upon its loss. In the forebrain, miR-9 is expressed in progenitors as well as developing neurons. Here, loss of miR-9 induces p53-mediated apoptosis, which counteracts the increase in proliferation leading to an unexpected reduction in the total number of neural progenitors (Bonev et al. 2011, 2012). This regional specificity might explain the so far contradictory data gathered on the function of miR-9 with regard to neural progenitor expansion (Zhao et al. 2009; Delaloy et al. 2010; Shibata et al. 2011).

The spatial identity of neural progenitors along the DV axis is also influenced by miRNAs. MiR-7a regulates adult neurogenesis in the olfactory bulb and is expressed in a dorso-ventral gradient in the ventricle walls (de Chevigny et al. 2012). Based on the segmentation of the lateral ventricle walls, defined types of olfactory bulb neurons are generated. Dopaminergic neurons are predominantly generated from progenitors located in the dorsal periventricular zone–a region exhibiting relatively low miR-7a expression (de Chevigny et al. 2012). Dopaminergic specification depends on the transcription factor Pax6, whose 3’UTR carries a functional binding site for miR-7a. Inhibition of miR-7a leads to an increased ventral Pax6 expression and a higher rate of differentiated dopaminergic neurons in the olfactory bulb (de Chevigny et al. 2012).

Recently, it was shown that miR-135a delimits the dorso-ventral extent of dopaminergic progenitors by targeting *Lmx1b* during murine midbrain development (Anderegg et al. 2013). The *FoxA2/Lmx1a/b* expression domain was markedly reduced upon ectopic expression of miR-135a–concomitantly with an impaired generation of TH-positive dopaminergic neurons. Furthermore, the dimension of the *Wnt1* expression domain and the overall Wnt activity in the developing midbrain were reduced. Overexpression of *Lmx1b* induced opposite effects, in that the midbrain dopaminergic progenitor domain was expanded and Wnt activity was increased. Interestingly, ectopic expression of *Lmx1b* increased miR-135a, while depletion of *Lmx1b* decreased the expression of this miRNA. Thus, miR-135a and Lmx1b might be engaged in a negative feed-back loop in fine-tuning Wnt activity, midbrain progenitor allocation and midbrain size. The impact of miR-135a on Wnt signaling might be in part mediated by the induced down-regulation of *Lmx1b*. However, miR-135a might also interfere directly with Wnt signaling, since several Wnt molecules were identified as potential miR-135a targets (Anderegg et al. 2013).

Together, these data illustrate how neural identities along the spatial coordinates within the nervous system are modulated by miRNAs fine-tuning the expression of important fate determinants and modulating morphogen signaling.

### MicroRNAs regulating temporal fate specification of neural progenitors

During retinal and cerebral cortex development, neural progenitor cells (retinal progenitors and radial glial cells, respectively) proceed through different competence states. This results in the successive emergence of distinct neuronal cell types, which are organized in a laminar pattern according to their neuronal birth order (corticogenesis: reviewed by Greig et al. 2013; retinogenesis: reviewed by Centanin and Wittbrodt 2013). The shift in progenitor competence over time is controlled by several transcription factors and there is mounting evidence that miRNAs play a regulatory role as well (reviewed by Cremisi 2013). Conditional deletion of Dicer during early mouse retinal development resulted in increased and prolonged production of early-born ganglion cells, while the production of late-born cell types was impaired (Georgi and Reh 2010). Similarly, Dicer-null mouse cortical stem cells were only able to produce early-born deep layer projection neurons and failed to generate late-born upper layer neurons (Saurat et al. 2013). The authors of this study proposed that the production of the late-born neurons might critically depend on an active miRNA system. Noteworthy to mention in this context is that the production of Cajal Retzius cells, which emit instructive cues for proper cortical development, is impaired in miR-9_2/9_3 double knock-out mice (Shibata et al. 2011). Accordingly, inhibition of miR-9 using an antisense oligonucleotide resulted in an abnormal development of cortical layers (Shibata et al. 2008, 2011). For more information on the impact of miR-9 during cortical neurogenesis, please refer to the review by Abernathy and Yoo in this Special Issue.

During retinal development, a specific subset of miRNAs has been shown to modulate the fate of neural progenitors by ensuring the correct temporal expression of key transcription factors (reviewed by Cremisi 2013). In *Xenopus*, the homeobox genes *Xotx2* and *Xvsx1* are necessary for the generation of bipolar neurons, the last neuronal cell type produced. Although the respective transcripts are already present in early retinal progenitors, Xotx2 and Xvsx1 protein is only detected at later stages due to the action of four miRNAs. These miRNAs, i.e., miR-129, miR-155, miR-214 and miR-222, are down-regulated during retinal development allowing the translation of *Xotx2* and *Xvsx1* (Decembrini et al. 2009). Inhibition of these miRNAs by transfection of respective antisense oligonucleotides into the optic vesicle resulted in an ectopic generation of bipolar neurons. Interestingly, their expression level is coupled to progenitor cell cycle length, which increases during retinal development and might serve as an intrinsic timer of neural progenitor age (Ohnuma et al. 2002; Decembrini et al. 2009; Pitto and Cremisi 2010). Lengthening of the cell cycle by inhibition of SHH signaling resulted in a decreased expression of miR-129, miR-155, miR-214 and miR-222, while speeding-up of cell cycle progression had the opposite effect (Decembrini et al. 2009). Another group of miRNAs, i.e., let-7, miR-125 and miR-9 has been shown to promote the progression of murine retinal progenitors from early to late fates via targeting *Protogenin (Ptrg)* and *Lin28b*, two factors involved in early retinal progenitor competence (La Torre et al. 2013). Overexpression of let-7, miR-125 and miR-9 accelerated progenitor fate progression and development of late-born neurons. In contrast, overexpression of their target genes–*Ptrg* and *Lin28b*—retained the progenitor cells at an early competence state. Similar to their function as developmental indicators of retinogenesis, recent findings have pointed to a role of let-7 and miR-125 during temporal fate specification in *Drosophila* mushroom body (MB) neurons (Wu et al. 2012; Kucherenko et al. 2012). Here, let-7 and miR-125 contribute to the progressive down-regulation of *chinmo*, which controls MB subtypes specification in a concentration-dependent manner (Wu et al. 2012). In addition to *chinmo*, let-7 and miR-125 also target *abrupt*, another temporal regulator of MB subtype specification (Kucherenko et al. 2012). Very recently, it was shown that olfactory bulb (OB) interneurons generated during embryogenesis show no miR-125b expression, while OB interneurons generated during adult neurogenesis exhibit miR-125b expression (Akerblom et al. 2014). Hence, the lack of miR-125b expression appears to distinguish OB interneuron subpopulations generated during different time periods suggesting that miR-125b might be implicated in regulating the temporal appearance of distinct neuronal subtypes. Interestingly, both let-7 and miR-125b regulate temporal fate progression of different lineages during *C. elegans* development (Olsen and Ambros 1999; Ambros et al. 2003; Ambros 2011). Together, these findings indicate that, although the cellular context is different, similar factors, such as let-7 and miR-125b, take part in controlling developmental fate transitions.

### MicroRNAs regulating neuronal subspecification in the spinal cord

Additional evidence for the importance of miRNAs as spatial and temporal regulators can be drawn from data on spinal cord development. The spinal cord, which is subdivided into (11) discrete neuronal progenitor domains along its dorso-ventral axis, is a well-characterized example of spatial neural patterning. Depending on the combinatorial transcription factor code expressed in the progenitors, each domain gives rise to a distinct set of neuronal subtypes, i.e., several classes of interneurons or motor neurons (MN) (reviewed by, e.g., Jessell 2000; Dessaud et al. 2008). Recently, three miRNAs (miR-17-3p, miR-196 and miR-9) have been shown to be involved in the subtype specification of spinal cord neurons.

The first example is miR-17-3p, which is implicated in the DV patterning of mouse spinal cord and affects motor neuron generation (Chen et al. 2011). Patterning of the developing spinal cord along the DV axis is mediated by the combinatorial action of SHH, retinoic acid (RA), BMP and Wnt signaling (reviewed by, e.g., Dessaud et al. 2008; Le Dréau and Martí 2012). These signals lead to the successive induction of key transcription factor determinants including pairs of transcriptional co-regulators. These pairs of cross-repressive transcription factors act as genetic switches in order to ensure unambiguous progenitor cell identity (Briscoe et al. 2000; Dessaud et al. 2010). The boundary between the motor neuron (pMN) and the V2 interneuron (p2) domain is specified by a cross-repressive loop between Olig2 and Irx3, the balance of which is regulated by miR-17-3p. *Olig2* is transiently expressed in the early p2 domain and subsequently down-regulated in order to allow *Irx3* expression and consolidation of p2 identity (Dessaud et al. 2010; Chen et al. 2011). MiR-17-3p is expressed in the p2 domain and represses *Olig2* by direct interaction with its 3’UTR. Loss of this miRNA resulted in an impaired production of V2 interneurons and an expansion of the pMN domain due to persistent *Olig2* expression in the p2 progenitors.

The post-mitotic motor neurons generated from the common Olig2-positive pMN domain are further diversified into different motor neuron subtypes, which are arranged in longitudinally oriented columns, i.e., median (M), hypaxial (H), preganglionic (P) and lateral (L) motor columns. The motor neuron columns are characterized by their unique axonal projection patterns to the musculature. For instance, the lateral motor column (LMC) innervates the muscles in the limb, whereas the median motor column (MMC) projects to axial muscles (Jessell 2000; Dasen and Jessell 2009; Philippidou and Dasen 2013). The lateral motor column (LMC) is further split into two subcolumns: the lateral LMCl and medial LMCm subcolumns. The definition of the different motor neuron identities is directed by Hox genes and accessory transcription factors, which themselves may be subject to miRNA-based regulation, e.g., by miR-196 and miR-9 (Yekta et al. 2008; Asli and Kessel 2010; Otaegi et al. 2011). During spinal cord development, miR-196 and *Hoxb8* exhibit a mutually exclusive expression pattern along the AP axis. MiR-196 was proposed to act as a post-transcriptional regulator of *Hoxb8* ensuring the absence of *Hoxb8* expression in the lumbar motor neuron segment (Asli and Kessel 2010). Ectopic expression of *Hoxb8* in the lumbar area resulted in an impaired generation of motor neurons. Although inhibition of miR-196 in the lumbar motor neuron column recapitulated the effect of *Hoxb8* overexpression, it did not lead to an up-regulation of Hoxb8 protein. Therefore, the authors argued that miR-196 might merely act as a fail-safe mechanism to prevent inappropriate *Hoxb8* expression secondary to the direct transcriptional regulation of the *Hoxb8* locus. A critical co-factor of Hox-dependent regulation of spatial motor neuron identities is FoxP1. Its expression is restricted to lateral (LMC) and preganglionic (PGC) motor neurons, which are—in contrast to hypoaxial (HMC) and medial (MMC) motor neurons–Hox-sensitive (Dasen and Jessell 2009). Mis-expression or depletion of *FoxP1* strongly affects motor neuron diversification and their columnar organization (Dasen et al. 2008; Rousso et al. 2008). In LMC, motor neurons of the chicken spinal cord levels of *FoxP1* are fine-tuned by overlapping miR-9 expression (Otaegi et al. 2011). Ectopic expression of miR-9 even switches LMC into MMC, thereby altering the targets of their axonal projections (Otaegi et al. 2011). This effect was counteracted by ectopic *FoxP1* expression further indicating that FoxP1 is an important target of miR-9 in MN subspecification (Otaegi et al. 2012). While electroporation of a competing *FoxP1* 3’UTR induced elevated FoxP1 and reduced HB9 levels, it did not alter the expression of MMC specific Lhx3 (Otaegi et al. 2011). However, a specific miR-9 sponge caused a mild reduction in Lhx3-positive neurons (Otaegi et al. 2012).

Besides the spatial regulation, the generation of the different motor neuron types from their common MN progenitor pool also depends on the temporal progenitor identity. Early progenitors give rise to MMC and early-born medial LMCm motor neurons followed by the production of late-born lateral LMCl motor neurons (Jessell 2000). The motor neurons originate from a unique progenitor pool that is diversified by transcription factors (Isl1, Lhx genes, OC1/Onecut1) as well as secreted molecules like retinoic acid (RA). RA is secreted by earlier-born motor neurons and was shown to induce the expression of specific miRNAs, including miR-9 (Kutty et al. 2010; Laneve et al. 2010). As recently shown, miR-9 might be involved in regulating the transition of progenitor competence from earlier-born to later-born motor neurons by targeting OC1 (Luxenhofer et al. 2014). Inhibition of miR-9 leads to increase of the OC1-positive earlier-born LMCm population resembling the mutually exclusive expression pattern of OC1 and miR-9 in the chick spinal cord (Luxenhofer et al. 2014). OC1 retains the expression of *Isl1* favoring the generation of earlier-born Isl1/FoxP1 double-positive motor neurons (Roy et al. 2012). MiR-9-mediated suppression of OC1 allows the induction of later-born motor neuron fate by relieving the Isl1-mediated repression of Lhx1. Therefore, earlier-born motor neurons were reduced upon ectopic miR-9 expression, while the rate of later-born motor neurons positive for Lhx1 and Hb9 was increased (Luxenhofer et al. 2014). Accordingly, a higher rate of earlier-born neurons was produced upon sponge-mediated miR-9 inhibition (Luxenhofer et al. 2014). These findings underline that the development of different neuronal subpopulations in the spinal cord depends on miRNA action in both spatial and temporal dimensions.

## MicroRNAs as tools to modulate cell fate and neuronal subtype decisions in vitro

The progress in stem cell research over the last two decades has opened new avenues for the generation of human neural cell types that were previously difficult to access (reviewed by, e.g., Koch et al. 2009a). Several protocols have been developed in order to direct the differentiation of pluripotent stem cells into distinct classes of neuronal cells. These approaches often rely on the usage of developmental signals known to confer certain neuronal identities in the developing CNS (for review see, e.g., Gaspard and Vanderhaeghen 2010; Petros et al. 2011; Peljto and Wichterle 2011; Tabar and Studer 2014). Considering that miRNAs are emerging as important players during in vivo neuronal subtype specification, they could be exploited as additional tools to modulate neuronal cell fate decisions in vitro (for an overview on the miRNAs identified to impact on neuronal subtype specification during in vitro differentiation paradigms, see Table 2). This was first shown by Kim et al. (2007), who reported a negative impact of miR-133b on the generation of dopaminergic neurons from mouse ES cells. A subset of midbrain dopaminergic neurons degenerate in Parkinson’s disease and are therefore of particular interest for neuro-regenerative stem cell research (reviewed by, e.g., Lindvall 2013; Arenas 2014). MiR-133b was found to be enriched in the human midbrain and depleted in the brain samples from Parkinson’s disease patients (Kim et al. 2007). Furthermore, expression of miR-133b was found to be induced by the dopaminergic transcription factor Pitx3. However, overexpression of miR-133b during ES cell differentiation or in primary midbrain cultures surprisingly impaired the generation of Tyrosine Hydroxylase (TH)-positive dopaminergic neurons. In line with that, inhibition of miR-133b resulted in an increased dopaminergic differentiation of mouse ES cells. Kim et al. (2007) further showed that miR-133b represses *Pitx3* expression via direct targeting and consequently hypothesized that miR-133b might regulate the maturation of dopaminergic neurons as part of a negative feed-back loop with *Pitx3*. However, it was later shown that miR-133b knock-out mice display normal dopaminergic neuron development (Heyer et al. 2012). A similar negative impact on the differentiation of dopaminergic neurons from mouse ES cells has been reported for miR-132 (Yang et al. 2012). Inhibition of this miRNA promoted the differentiation of TH-positive neurons, while overexpression of miR-132 had the opposite effect. The relevant miR-132 target in this context is the transcription factor *Nurr1*, which is an important regulator of dopaminergic differentiation. Using a TH promoter-driven GFP reporter, the authors could show that miR-132 is enriched in the GFP-positive cell population, which might be explained by the indirect inducing effect of Nurr1 on miR-132 expression (Yang et al. 2012). Nurr1 is known for its role as an activator of *BDNF* expression (Volpicelli et al. 2007), whereas BDNF itself was previously shown to induce miR-132 (Klein et al. 2007). Following this line of evidence, Yang et al. (2012) proposed that miR-132 might regulate dopaminergic differentiation as part of a feed-back loop with Nurr1 and BDNF. It is noteworthy to mention that bona fide midbrain dopaminergic neurons are characterized by the expression of a distinct set of markers and transcription factors (Smidt and Burbach 2007; Ono et al. 2007). Both studies on the impact of miR-133b and miR-132 described above solely determined the amount of TH-positive neurons, which might not be sufficient to reliably characterize dopaminergic neurons.

**Table 2:** MicroRNAs impacting on in vitro dopaminergic differentiation

miRNA	Target	Function	Cell-type	Reference
miR-133b	Pitx3	Inhibition of the generation of TH-positive neurons (no impact on DA neuron development in miR-133 knock-out mice)	Mouse ES cells	Kim et al. 2007; Heyer et al. 2012
miR-132	Nurr1	Inhibition of the generation of TH-positive neurons	Mouse ES cells	Yang et al. 2012
miR-181a, miR-125b	–	Promotion of the generation of TH-positive neurons	Human ES cel- derived lt-NES cells	Stappert et al. 2013
miR-181a*	–	Inhibition of the generation of TH-positive neurons		

*DA* dopaminergic, *TH* tyrosine hydroxylase

### Using human neural stem cells to study microRNAs in a human context

Most of the findings discussed above rely on experiments in animal model systems and may not always be transferrable to human neural cells (Gao 2009). In order to use miRNAs as tools to generate other neuronal cell types such as cortical, retinal and motor neuron subtypes, there is increasing interest to translate and extend the findings to in vitro differentiation protocols using human pluripotent stem cells (see also Benchoua and Peschanski 2013). However, the generation of mature neuronal cell types from hPS cells via so-called run-through protocols is prone to variability. Proliferative neural stem cells (NSCs) that can be derived from hPS cells as a stable intermediate might be used to minimize this variability. There are several protocols available to derive different NSC populations from hPS cells, such as primitive pre-rosette neuroepithelial stem cells (Li et al. 2011b; Reinhardt et al. 2013), rosette-forming neuroepithelial stem cells (Elkabetz et al. 2008; Koch et al. 2009b) and radial-glial like neural stem cells (Conti et al. 2005). These NSC populations can be distinguished by their morphology, self-renewal capacity and differentiation potential and are likely to represent different developmental stages similar to the range of NSCs generated in vivo. For a comparison of the different NSC populations please refer to the reviews by Conti and Cattaneo (2010) and Karus et al. (2014). Intriguingly, NSCs with similar properties have been successfully isolated from mouse (Hitoshi et al. 2004; Elkabetz et al. 2008) and even human brain (Tailor et al. 2013), indicating that these in vitro-generated NSCs might be a valuable model system for early neural development. Furthermore, given that NSC production and maintenance relies on inhibition of BMP/TGFβ signaling and activation of Wnt and Notch signaling among other signals (e.g., Borghese et al. 2010; Li et al. 2011b; Reinhardt et al. 2013), it is likely that miRNAs regulating these pathways (as discussed above) might also influence NSC fate. Hence, we and others used hPS cell-derived NSCs to first assess stage-dependent miRNA signatures during human neuronal differentiation, a topic that has been difficult to address due to the limited access to primary human neural tissue (Wu et al. 2007; Liu et al. 2012; Stappert et al. 2013). The identified miRNA expression patterns in many cases overlapped with data from previous miRNA profiling analyses performed in rodent models, mouse ES cells or immortalized cell lines (Sempere et al. 2004; Krichevsky et al. 2006; Smith et al. 2010), indicating that many miRNA functions might be conserved between species.

**Fig. 3:**
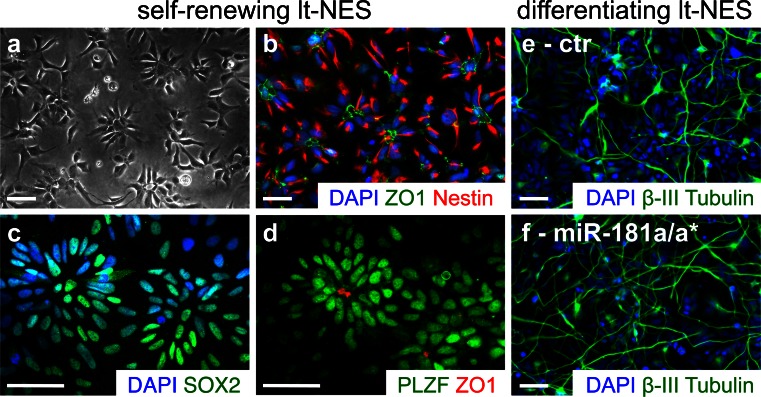
Lt-NES cells can be used to study miRNA functions associated with human neuronal differentiation. (**a-d**) Self-renewing lt-NES cell form small neural rosettes with characteristic ZO1 expression in the lumen (**b**, **d**). They express the neural stem cell markers Nestin (**b**), SOX2 (**c**) and PLZF (**d**). (**e**) When induced to enter differentiation by growth factor withdrawal, lt-NES cells give rise to β-III tubulin-positive neurons as shown here after 7 days of differentiation. (**f**) The rate of neuronal differentiation can be further increased by lentivirus-mediated overexpression of neuronal fate-associated miRNAs such as miR-181a/a*. *Ctr* lt-NES cell cultures transduced with a control lentiviral construct coding for a scrambled miRNA. *DAPI* labels nuclei, all *scale bars* 50 μm. The pictures in (**c**, **d**) were kindly provided by Johannes Jungverdorben

In order to identify novel miRNA functions associated with human neuronal differentiation, we took advantage of a population of long-term self-renewing neuroepithelial-like stem cells (lt-NES) developed in our institute (Koch et al. 2009b; Falk et al. 2012). These cells show an extensive self-renewal capacity when cultured in the presence of FGF2, EGF and low concentrations of B27 cell culture supplement but also retain a stable neurogenic differentiation potential. Self-renewing lt-NES cells arrange in small neural rosette structures, which are characterized by expression of the tight-junction protein ZO1 (TJP1) in the rosette lumen (Fig. 3a–d). They are also positive for NSCs markers such as Nestin, SOX2 and PLZF (ZBTB16) and, according to their transcription expression factor profile, display an anterior hindbrain identity. After growth factor withdrawal, lt-NES cells differentiate primarily into neurons, marked by the expression of the pan-neuronal marker β-III tubulin (Fig. 3e). After prolonged differentiation, they also give rise to astrocytes as well as a few oligodendrocytes. Intriguingly, cells with similar properties have recently been generated from embryonic human hindbrain specimens, indicating that lt-NES cells do not just represent an artifact of hPS cell in vitro differentiation (Tailor et al. 2013). Lt-NES cells have been successfully used to model human neurodegenerative diseases (Koch et al. 2011, 2012) and for screening and validating pharmacological compounds (McLaren et al. 2013; Mertens et al. 2013). Lt-NES cells are amenable to stable and transient miRNA modification and respond well to known neuronal fate-associated miR-124, miR-125b and miR-9/9* (Stappert et al. 2013; Roese-Koerner et al. 2013). In a proof-of-principle experiment, we showed that overexpression of the respective miRNA loci encoding these miRNAs promotes neuronal differentiation of lt-NES cells. We further showed that the two miRNAs produced from the bifunctional miR-9/9* loci have a divergent impact on lt-NES cells. Individual modulation of both miR-9 and miR-9* activities revealed that both miRNAs promote neuronal differentiation of lt-NES cells, while only miR-9* was found to inhibit cell proliferation (Roese-Koerner et al. 2013). These findings might point to an even more complex function of miR-9/9* within the gene regulatory networks controlling proliferation and neuronal differentiation. Recently, we identified three additional miRNAs, i.e., miR-153, miR-181a/a* and miR-324-5p/3p, which promote neuronal differentiation (Stappert et al. 2013; see also Fig. 3f).

With regard to neuronal subtype specification, we identified two miRNAs that promote the generation of dopamine-like neurons from lt-NES cells (Stappert et al. 2013). Lt-NES cells have a strong differentiation bias to GABAergic neurons. However, they can also give rise to other neuronal phenotypes, such as motor neurons and dopaminergic neurons, when cultured in the presence of patterning cues. For instance, culturing lt-NES cells in the presence of SHH and FGF8b, two morphogenes that are important for dopaminergic neuron specification (Ye et al. 1998), leads to the generation of TH-positive dopamine-like neurons (Koch et al. 2009b; Falk et al. 2012). Furthermore, neuronal subtype specification of differentiating lt-NES cells may be influenced by specific miRNAs (Stappert et al. 2013). By gain- and loss-of-function experiments we could show that miR-181a and miR-125b specifically promote the emergence of TH-positive dopamine-like neurons from lt-NES cells. Interestingly, miR-181a* inhibited the formation of this neuronal population, indicating an intrinsic regulatory mechanism of the bifunctional miR-181a/a* on dopaminergic differentiation. This might also be reflected by the expression levels of miR-181a versus miR-181a*, the ratio of which is increased in human fetal midbrain compared to human whole fetal brain extracts. Moreover, transient delivery of the respective miRNA mimics and inhibitors was sufficient to affect neuronal subtype specification of lt-NES cells and could thus be used to augment the treatment with patterning signals. Thus, it would be interesting to combine miRNA modulation with the recently improved protocols specifically tailored towards efficient generation of midbrain dopaminergic neurons from human pluripotent stem cells (Kriks et al. 2011; Kirkeby et al. 2012; Xi et al. 2012).

The generation of authentic neuronal cell types that fully resemble their in vivo counterparts is still one of the main challenges in stem cell research. For some neuronal cell types, it is even unclear which markers are required to demonstrate the authenticity of the desired cell types. This problem is further aggravated by the fact that the overall knowledge on the gene regulatory circuitries contributing to neuronal diversification and neuronal transmitter-specific functions is rather limited (reviewed by, e.g., Ernsberger 2012; Sandoe and Eggan 2013). The data discussed above demonstrate that miRNAs play important roles in regulating neural differentiation and conferring neuronal identities and should therefore be taken into consideration when annotating neuronal subtype-specific marker gene expression profiles. In this context, subtype-specific reporter cell lines and RNA sequencing analysis could be used to assess the coding and non-coding transcriptome of specific neuronal cell types on a global scale. This information could, in the end, be harnessed to further refine in vitro differentiation paradigms.

## Conclusions

Recent findings have placed miRNAs in the midst of gene regulatory networks involved in neural induction, neuronal differentiation and fate specification. MicroRNAs contribute to the establishment of transcriptional codes determining the ground-state of cellular identity. However, knowledge on the impact of miRNA-based regulation during human neural development is still limited–a gap that could be closed by the increasing availability of human neural cell types generated from human pluripotent stem cells. In this context, well-defined populations of human neural stem cells, such as lt-NES cells, could be used to study miRNAs with regard to early human neural development. As indicated by a few pioneer studies, miRNAs could be envisioned as tools to direct the differentiation of pluripotent stem cells and derived neural stem cells towards medically relevant neuronal subtypes. Each miRNA may have numerous mRNA targets and modulating a single miRNA may thus alter the entire differentiation process, making miRNA-based regulation an attractive approach for in vitro specification of neuronal cell fates. Furthermore, miRNA activity may be transiently modulated by applying synthetic miRNA mimics and inhibitors, which could be easily combined with other patterning cues. Finally, deregulation of miRNA activity is associated with many neurodegenerative diseases. Therefore, miRNAs may represent promising targets to develop novel therapeutic approaches (reviewed by, e.g., Junn and Mouradian 2012; Maciotta et al. 2013), whose potential might be evaluated using the iPS cell technology. Altogether, connecting miRNAs to specific functions during human neural development has a great value for the deeper understanding of both physiological and pathological processes in the CNS.

## References

[CR1] Akerblom M, Jakobsson J (2013). MicroRNAs as neuronal fate determinants. Neuroscientist.

[CR2] Akerblom M, Sachdeva R, Jakobsson J (2012). Functional studies of microRNAs in neural stem cells: problems and perspectives. Front Neurosci.

[CR3] Akerblom M, Petri R, Sachdeva R, Klussendorf T, Mattsson B, Gentner B, Jakobsson J (2014) microRNA-125 distinguishes developmentally generated and adult-born olfactory bulb interneurons. Development. doi: 10.1242/dev.101659 10.1242/dev.101659 24598163

[CR4] Alqadah A, Hsieh Y-W, Chuang C-F (2013) MicroRNA function in left-right neuronal asymmetry: perspectives from C. elegans. Front Cell Neurosci 7:158 10.3389/fncel.2013.00158 PMC3779813 24065887

[CR5] Ambasudhan R, Talantova M, Coleman R, Yuan X, Zhu S, Lipton SA, Ding S (2011). Direct reprogramming of adult human fibroblasts to functional neurons under defined conditions. Cell Stem Cell.

[CR6] Ambros V (2011). MicroRNAs and developmental timing. Curr Opin Genet Dev.

[CR7] Ambros V, Lee RC, Lavanway A, Williams PT, Jewell D (2003). MicroRNAs and other tiny endogenous RNAs in C. elegans. Curr Biol.

[CR8] Anderegg A, Lin H-P, Chen J-A, Caronia-Brown G, Cherepanova N, Yun B, Joksimovic M, Rock J, Harfe BD, Johnson R, Awatramani R (2013). An Lmx1b-miR135a2 regulatory circuit modulates Wnt1/Wnt signaling and determines the size of the midbrain dopaminergic progenitor pool. PLoS Genet.

[CR9] Aoki H, Hara A, Era T, Kunisada T, Yamada Y (2012). Genetic ablation of Rest leads to in vitro-specific derepression of neuronal genes during neurogenesis. Development.

[CR10] Arenas E (2014). Wnt signaling in midbrain dopaminergic neuron development and regenerative medicine for Parkinson’s disease. J Mol Cell Biol.

[CR11] Arora S, Rana R, Chhabra A, Jaiswal A, Rani V (2013). miRNA–transcription factor interactions: a combinatorial regulation of gene expression. Mol Genet Genomics.

[CR12] Asli NS, Kessel M (2010). Spatiotemporally restricted regulation of generic motor neuron programs by miR-196-mediated repression of Hoxb8. Dev Biol.

[CR13] Benchoua A, Peschanski M (2013). Pluripotent stem cells as a model to study non-coding RNAs function in human neurogenesis. Front Cell Neurosci.

[CR14] Bhinge A, Poschmann J, Namboori SC, Tian X, Jia Hui Loh S, Traczyk A, Prabhakar S, Stanton LW (2014). MiR-135b is a direct PAX6 target and specifies human neuroectoderm by inhibiting TGF-β/BMP signaling. EMBO J.

[CR15] Bian S, Xu T-L, Sun T (2013). Tuning the cell fate of neurons and glia by microRNAs. Curr Opin Neurobiol.

[CR16] Biryukova I, Asmar J, Abdesselem H, Heitzler P (2009). Drosophila mir-9a regulates wing development via fine-tuning expression of the LIM only factor, dLMO. Dev Biol.

[CR17] Boissart C, Nissan X, Giraud-Triboult K, Peschanski M, Benchoua A (2012). miR-125 potentiates early neural specification of human embryonic stem cells. Development.

[CR18] Bonev B, Pisco A, Papalopulu N (2011). MicroRNA-9 reveals regional diversity of neural progenitors along the anterior-posterior axis. Dev Cell.

[CR19] Bonev B, Stanley P, Papalopulu N (2012). MicroRNA-9 modulates Hes1 ultradian oscillations by forming a double-negative feedback loop. Cell Rep.

[CR20] Borghese L, Dolezalova D, Opitz T, Haupt S, Leinhaas A, Steinfarz B, Koch P, Edenhofer F, Hampl A, Brüstle O (2010). Inhibition of Notch signaling in human embryonic stem cell-derived neural stem cells delays G1/S phase transition and accelerates neuronal differentiation in vitro and in vivo. Stem Cells.

[CR21] Briscoe J, Pierani A, Jessell TM, Ericson J (2000). A homeodomain protein code specifies progenitor cell identity and neuronal fate in the ventral neural tube. Cell.

[CR22] Burk U, Schubert J, Wellner U, Schmalhofer O, Vincan E, Spaderna S, Brabletz T (2008). A reciprocal repression between ZEB1 and members of the miR-200 family promotes EMT and invasion in cancer cells. EMBO Rep.

[CR23] Cassidy JJ, Jha AR, Posadas DM, Giri R, Venken KJT, Ji J, Jiang H, Bellen HJ, White KP, Carthew RW (2013). miR-9a minimizes the phenotypic impact of genomic diversity by buffering a transcription factor. Cell.

[CR24] Centanin L, Wittbrodt J (2013). Retinal neurogenesis. Development.

[CR25] Chambers SM, Fasano CA, Papapetrou EP, Tomishima M, Sadelain M, Studer L (2009). Highly efficient neural conversion of human ES and iPS cells by dual inhibition of SMAD signaling. Nat Biotechnol.

[CR26] Chen J-A, Wichterle H (2012). Apoptosis of limb innervating motor neurons and erosion of motor pool identity upon lineage specific dicer inactivation. Front Neurosci.

[CR27] Chen J-A, Huang Y-P, Mazzoni EO, Tan GC, Zavadil J, Wichterle H (2011). Mir-17-3p controls spinal neural progenitor patterning by regulating Olig2/Irx3 cross-repressive loop. Neuron.

[CR28] Cheng L-C, Pastrana E, Tavazoie M, Doetsch F (2009). miR-124 regulates adult neurogenesis in the subventricular zone stem cell niche. Nat Neurosci.

[CR29] Conaco C, Otto S, Han J-JJ, Mandel G (2006). Reciprocal actions of REST and a microRNA promote neuronal identity. Proc Natl Acad Sci U S A.

[CR30] Conti L, Cattaneo E (2010). Neural stem cell systems: physiological players or in vitro entities?. Nat Rev Neurosci.

[CR31] Conti L, Pollard SM, Gorba T, Reitano E, Toselli M, Biella G, Sun Y, Sanzone S, Ying Q-L, Cattaneo E, Smith A (2005). Niche-independent symmetrical self-renewal of a mammalian tissue stem cell. PLoS Biol.

[CR32] Coolen M, Thieffry D, Drivenes Ø, Becker TS, Bally-Cuif L (2012). miR-9 controls the timing of neurogenesis through the direct inhibition of antagonistic factors. Dev Cell.

[CR33] Coolen M, Katz S, Bally-Cuif L (2013) miR-9: a versatile regulator of neurogenesis. Front Cell Neurosci 7:220 10.3389/fncel.2013.00220 PMC3834235 24312010

[CR34] Cremisi F (2013). MicroRNAs and cell fate in cortical and retinal development. Front Cell Neurosci.

[CR35] Dasen JS, Jessell TM (2009) Hox networks and the origins of motor neuron diversity. Curr Top Dev Biol 88:169–200 10.1016/S0070-2153(09)88006-X 19651305

[CR36] Dasen JS, De Camilli A, Wang B, Tucker PW, Jessell TM (2008). Hox repertoires for motor neuron diversity and connectivity gated by a single accessory factor, FoxP1. Cell.

[CR37] Davis TH, Cuellar TL, Koch SM, Barker AJ, Harfe BD, McManus MT, Ullian EM (2008). Conditional loss of Dicer disrupts cellular and tissue morphogenesis in the cortex and hippocampus. J Neurosci.

[CR38] de Chevigny A, Coré N, Follert P, Gaudin M, Barbry P, Béclin C, Cremer H (2012). miR-7a regulation of Pax6 controls spatial origin of forebrain dopaminergic neurons. Nat Neurosci.

[CR39] De Pietri TD, Pulvers JN, Haffner C, Murchison EP, Hannon GJ, Huttner WB (2008). miRNAs are essential for survival and differentiation of newborn neurons but not for expansion of neural progenitors during early neurogenesis in the mouse embryonic neocortex. Development.

[CR40] Decembrini S, Bressan D, Vignali R, Pitto L, Mariotti S, Rainaldi G, Wang X, Evangelista M, Barsacchi G, Cremisi F (2009). MicroRNAs couple cell fate and developmental timing in retina. Proc Natl Acad Sci U S A.

[CR41] Delaloy C, Liu L, Lee JA, Su H, Shen F, Yang GY, Young WL, Ivey KN, Gao FB (2010). MicroRNA-9 coordinates proliferation and migration of human embryonic stem cell-derived neural progenitors. Cell Stem Cell.

[CR42] Dessaud E, McMahon AP, Briscoe J (2008). Pattern formation in the vertebrate neural tube: a sonic hedgehog morphogen-regulated transcriptional network. Development.

[CR43] Dessaud E, Ribes V, Balaskas N, Yang LL, Pierani A, Kicheva A, Novitch BG, Briscoe J, Sasai N (2010). Dynamic assignment and maintenance of positional identity in the ventral neural tube by the morphogen sonic hedgehog. PLoS Biol.

[CR44] Du ZW, Ma LX, Phillips C, Zhang SC (2013). miR-200 and miR-96 families repress neural induction from human embryonic stem cells. Development.

[CR45] Elkabetz Y, Panagiotakos G, Shamy Al G, Socci ND, Tabar V, Studer L (2008). Human ES cell-derived neural rosettes reveal a functionally distinct early neural stem cell stage. Genes Dev.

[CR46] Ernsberger U (2012). Regulation of gene expression during early neuronal differentiation: evidence for patterns conserved across neuron populations and vertebrate classes. Cell Tissue Res.

[CR47] Esteller M (2011). Non-coding RNAs in human disease. Nat Rev Genet.

[CR48] Falk A, Koch P, Kesavan J, Takashima Y, Ladewig J, Alexander M, Wiskow O, Tailor J, Trotter M, Pollard S, Smith A, Brüstle O (2012). Capture of neuroepithelial-like stem cells from pluripotent stem cells provides a versatile system for in vitro production of human neurons. PLoS ONE.

[CR49] Fishell G, Heintz N (2013). The neuron identity problem: form meets function. Neuron.

[CR50] Gao F-B (2009). Context-dependent functions of specific microRNAs in neuronal development. Neural Dev.

[CR51] Gaspard N, Vanderhaeghen P (2010). Mechanisms of neural specification from embryonic stem cells. Curr Opin Neurobiol.

[CR52] Gaughwin P, Ciesla M, Yang H, Lim B, Brundin P (2011). Stage-specific modulation of cortical neuronal development by mmu-miR-134. Cereb Cortex.

[CR53] Georgi SA, Reh TA (2010). Dicer is required for the transition from early to late progenitor state in the developing mouse retina. J Neurosci.

[CR54] Giraldez AJ, Cinalli RM, Glasner ME, Enright AJ, Thomson JM, Baskerville S, Hammond SM, Bartel DP, Schier AF (2005). MicroRNAs regulate brain morphogenesis in zebrafish. Science.

[CR55] Greig LC, Woodworth MB, Galazo MJ, Padmanabhan H, Macklis JD (2013). Molecular logic of neocortical projection neuron specification, development and diversity. Nat Rev Neurosci.

[CR56] Greve TS, Judson RL, Blelloch R (2013). MicroRNA control of mouse and human pluripotent stem cell behavior. Annu Rev Cell Dev Biol.

[CR57] Guo Y, Chen Y, Ito H, Watanabe A, Ge X, Kodama T, Aburatani H (2006). Identification and characterization of lin-28 homolog B (LIN28B) in human hepatocellular carcinoma. Gene.

[CR58] He M, Liu Y, Wang X, Zhang MQ, Hannon GJ, Huang ZJ (2012). Cell-type-based analysis of microRNA profiles in the mouse brain. Neuron.

[CR59] He X, Yu Y, Awatramani R, Lu QR (2012). Unwrapping myelination by microRNAs. Neuroscientist.

[CR60] Heo I, Joo C, Kim YK, Ha M, Yoon MJ, Cho J, Yeom KH, Han J, Kim VN (2009). TUT4 in concert with Lin28 suppresses microRNA biogenesis through pre-microRNA uridylation. Cell.

[CR61] Herranz H, Cohen SM (2010). MicroRNAs and gene regulatory networks: managing the impact of noise in biological systems. Genes Dev.

[CR62] Heyer MP, Pani AK, Smeyne RJ, Kenny PJ, Feng G (2012). Normal midbrain dopaminergic neuron development and function in miR-133b mutant mice. J Neurosci.

[CR63] Hitoshi S, Seaberg RM, Koscik C, Alexson T, Kusunoki S, Kanazawa I, Tsuji S, van der Kooy D (2004). Primitive neural stem cells from the mammalian epiblast differentiate to definitive neural stem cells under the control of Notch signaling. Genes Dev.

[CR64] Hobert O (2004). Common logic of transcription factor and microRNA action. Trends Biochem Sci.

[CR65] Huang T, Liu Y, Huang M, Zhao X, Cheng L (2010). Wnt1-cre-mediated conditional loss of Dicer results in malformation of the midbrain and cerebellum and failure of neural crest and dopaminergic differentiation in mice. J Mol Cell Biol.

[CR66] Inui M, Martello G, Piccolo S (2010). MicroRNA control of signal transduction. Nat Rev Mol Cell Biol.

[CR67] Inui M, Montagner M, Piccolo S (2012). miRNAs and morphogen gradients. Curr Opin Cell Biol.

[CR68] Jessell TM (2000). Neuronal specification in the spinal cord: inductive signals and transcriptional codes. Nat Rev Genet.

[CR69] Johnston RJ, Hobert O (2003). A microRNA controlling left/right neuronal asymmetry in Caenorhabditis elegans. Nature.

[CR70] Johnston RJ, Chang S, Etchberger JF, Ortiz CO, Hobert O (2005). MicroRNAs acting in a double-negative feedback loop to control a neuronal cell fate decision. Proc Natl Acad Sci U S A.

[CR71] Junn E, Mouradian MM (2012). MicroRNAs in neurodegenerative diseases and their therapeutic potential. Pharmacol Ther.

[CR72] Kanellopoulou C, Muljo SA, Kung AL, Ganesan S, Drapkin R, Jenuwein T, Livingston DM, Rajewsky K (2005). Dicer-deficient mouse embryonic stem cells are defective in differentiation and centromeric silencing. Genes Dev.

[CR73] Kapsimali M, Kloosterman WP, de Bruijn E, Rosa F, Plasterk RH, Wilson SW (2007). MicroRNAs show a wide diversity of expression profiles in the developing and mature central nervous system. Genome Biol.

[CR74] Karus M, Blaess S, Brüstle O (2014). Self-organisation of neural tissue architectures from pluripotent stem cells. J Comp Neurol.

[CR75] Kiecker C, Lumsden A (2012). The role of organizers in patterning the nervous system. Annu Rev Neurosci.

[CR76] Kim J, Inoue K, Ishii J, Vanti WB, Voronov SV, Murchison E, Hannon G, Abeliovich A (2007). A MicroRNA feedback circuit in midbrain dopamine neurons. Science.

[CR77] Kim H, Lee G, Ganat Y, Papapetrou EP, Lipchina I, Socci ND, Sadelain M, Studer L (2011). miR-371-3 expression predicts neural differentiation propensity in human pluripotent stem cells. Cell Stem Cell.

[CR78] Kirkeby A, Grealish S, Wolf DA, Nelander J, Wood J, Lundblad M, Lindvall O, Parmar M (2012). Generation of regionally specified neural progenitors and functional neurons from human embryonic stem cells under defined conditions. Cell Rep.

[CR79] Klein ME, Lioy DT, Ma L, Impey S, Mandel G, Goodman RH (2007). Homeostatic regulation of MeCP2 expression by a CREB-induced microRNA. Nat Neurosci.

[CR80] Koch P, Kokaia Z, Lindvall O, Brüstle O (2009). Emerging concepts in neural stem cell research: autologous repair and cell-based disease modelling. Lancet Neurol.

[CR81] Koch P, Opitz T, Steinbeck JA, Ladewig J, Brüstle O (2009). A rosette-type, self-renewing human ES cell-derived neural stem cell with potential for in vitro instruction and synaptic integration. Proc Natl Acad Sci U S A.

[CR82] Koch P, Breuer P, Peitz M, Jungverdorben J, Kesavan J, Poppe D, Doerr J, Ladewig J, Mertens J, Tüting T, Hoffmann P, Klockgether T, Evert BO, Wüllner U, Brüstle O (2011). Excitation-induced ataxin-3 aggregation in neurons from patients with Machado-Joseph disease. Nature.

[CR83] Koch P, Tamboli IY, Mertens J, Wunderlich P, Ladewig J, Stuber K, Esselmann H, Wiltfang J, Brüstle O, Walter J (2012). Presenilin-1 L166P mutant human pluripotent stem cell-derived neurons exhibit partial loss of gamma-secretase activity in endogenous amyloid-beta generation. Am J Pathol.

[CR84] Kohwi M, Doe CQ (2013). Temporal fate specification and neural progenitor competence during development. Nat Rev Neurosci.

[CR85] Kozomara A, Griffiths-Jones S (2011). miRBase: integrating microRNA annotation and deep-sequencing data. Nucleic Acids Res.

[CR86] Kozomara A, Griffiths-Jones S (2013). miRBase: annotating high confidence microRNAs using deep sequencing data. Nucleic Acids Res.

[CR87] Krichevsky AM, King KS, Donahue CP, Khrapko K, Kosik KS (2003). A microRNA array reveals extensive regulation of microRNAs during brain development. RNA.

[CR88] Krichevsky AM, Sonntag K-C, Isacson O, Kosik KS (2006). Specific microRNAs modulate embryonic stem cell-derived neurogenesis. Stem Cells.

[CR89] Kriks S, Shim J-W, Piao J, Ganat YM, Wakeman DR, Xie Z, Carrillo-Reid L, Auyeung G, Antonacci C, Buch A, Yang L, Beal MF, Surmeier DJ, Kordower JH, Tabar V, Studer L (2011). Dopamine neurons derived from human ES cells efficiently engraft in animal models of Parkinson’s disease. Nature.

[CR90] Kucherenko MM, Barth J, Fiala A, Shcherbata HR (2012). Steroid-induced microRNA let-7 acts as a spatio-temporal code for neuronal cell fate in the developing Drosophila brain. EMBO J.

[CR91] Kutty RK, Samuel W, Jaworski C, Duncan T, Nagineni CN, Raghavachari N, Wiggert B, Redmond TM (2010). MicroRNA expression in human retinal pigment epithelial (ARPE-19) cells: increased expression of microRNA-9 by N-(4-hydroxyphenyl) retinamide. Mol Vis.

[CR92] La Torre A, Georgi S, Reh TA (2013). Conserved microRNA pathway regulates developmental timing of retinal neurogenesis. Proc Natl Acad Sci U S A.

[CR93] Landgraf P, Rusu M, Sheridan R, Sewer A, Iovino N, Aravin A, Pfeffer S, Rice A, Kamphorst AO, Landthaler M, Lin C, Socci ND, Hermida L, Fulci V, Chiaretti S, Foa R, Schliwka J, Fuchs U, Novosel A, Muller RU, Schermer B, Bissels U, Inman J, Phan Q, Chien M, Weir DB, Choksi R, De Vita G, Frezzetti D, Trompeter HI, Hornung V, Teng G, Hartmann G, Palkovits M, Di Lauro R, Wernet P, Macino G, Rogler CE, Nagle JW, Ju J, Papavasiliou FN, Benzing T, Lichter P, Tam W, Brownstein MJ, Bosio A, Borkhardt A, Russo JJ, Sander C, Zavolan M, Tuschl T (2007). A mammalian microRNA expression atlas based on small RNA library sequencing. Cell.

[CR94] Laneve P, Gioia U, Andriotto A, Moretti F, Bozzoni I, Caffarelli E (2010). A minicircuitry involving REST and CREB controls miR-9-2 expression during human neuronal differentiation. Nucleic Acids Res.

[CR95] Le Dréau G, Martí E (2012). Dorsal-ventral patterning of the neural tube: A tale of three signals. Devel Neurobio.

[CR96] Lee H, Shamy GA, Elkabetz Y, Schofield CM, Harrsion NL, Panagiotakos G, Socci ND, Tabar V, Studer L (2007). Directed differentiation and transplantation of human embryonic stem cell-derived motoneurons. Stem Cells.

[CR97] Leucht C, Stigloher C, Wizenmann A, Klafke R, Folchert A, Bally-Cuif L (2008). MicroRNA-9 directs late organizer activity of the midbrain-hindbrain boundary. Nat Neurosci.

[CR98] Lewis BP, Burge CB, Bartel DP (2005). Conserved seed pairing, often flanked by adenosines, indicates that thousands of human genes are microRNA targets. Cell.

[CR99] Li Y, Wang F, Lee JA, Gao FB (2006). MicroRNA-9a ensures the precise specification of sensory organ precursors in Drosophila. Genes Dev.

[CR100] Li Q, Bian S, Hong J, Kawase-Koga Y, Zhu E, Zheng Y, Yang L, Sun T (2011). Timing specific requirement of microRNA function is essential for embryonic and postnatal hippocampal development. PLoS ONE.

[CR101] Li W, Sun W, Zhang Y, Wei W, Ambasudhan R, Xia P, Talantova M, Lin T, Kim J, Wang X, Kim WR, Lipton SA, Zhang K, Ding S (2011). Rapid induction and long-term self-renewal of primitive neural precursors from human embryonic stem cells by small molecule inhibitors. Proc Natl Acad Sci U S A.

[CR102] Lim LP, Lau NC, Garrett-Engele P, Grimson A, Schelter JM, Castle J, Bartel DP, Linsley PS, Johnson JM (2005). Microarray analysis shows that some microRNAs downregulate large numbers of target mRNAs. Nature.

[CR103] Lindvall O (2013). Developing dopaminergic cell therapy for Parkinson’s disease-give up or move forward?. Mov Disord.

[CR104] Lipchina I, Elkabetz Y, Hafner M, Sheridan R, Mihailovic A, Tuschl T, Sander C, Studer L, Betel D (2011). Genome-wide identification of microRNA targets in human ES cells reveals a role for miR-302 in modulating BMP response. Genes Dev.

[CR105] Liu XS, Chopp M, Zhang RL, Tao T, Wang XL, Kassis H, Hozeska-Solgot A, Zhang L, Chen C, Zhang ZG (2011). MicroRNA profiling in subventricular zone after stroke: miR-124a regulates proliferation of neural progenitor cells through Notch signaling pathway. PLoS ONE.

[CR106] Liu J, Githinji J, Mclaughlin B, Wilczek K, Nolta J (2012). Role of miRNAs in neuronal differentiation from human embryonic stem cell-derived neural stem cells. Stem Cell Rev Rep.

[CR107] Lumsden A, Krumlauf R (1996). Patterning the vertebrate neuraxis. Science.

[CR108] Luxenhofer G, Helmbrecht MS, Langhoff J, Giusti SA, Refojo D, Huber AB (2014). MicroRNA-9 promotes the switch from early-born to late-born motor neuron populations by regulating Onecut transcription factor expression. Dev Biol.

[CR109] Maciotta S, Meregalli M, Torrente Y (2013). The involvement of microRNAs in neurodegenerative diseases. Front Cell Neurosci.

[CR110] McLaren D, Gorba T, Marguerie de Rotrou A, Pillai G, Chappell C, Stacey A, Lingard S, Falk A, Smith A, Koch P, Brüstle O, Vickers R, Tinsley J, Flanders D, Bello P, Craig S (2013). Automated large-scale culture and medium-throughput chemical screen for modulators of proliferation and viability of human induced pluripotent stem cell-derived neuroepithelial-like stem cells. J Biomol Screen.

[CR111] McNeill E, Van Vactor D (2012). MicroRNAs shape the neuronal landscape. Neuron.

[CR112] Mertens J, Stüber K, Wunderlich P, Ladewig J, Kesavan JC, Vandenberghe R, Vandenbulcke M, van Damme P, Walter J, Brüstle O, Koch P (2013) APP processing in human pluripotent stem cell-derived neurons is resistant to NSAID-based γ-secretase modulation. Stem Cell Rep 2013:1–8 10.1016/j.stemcr.2013.10.011 PMC3871388 24371804

[CR113] Miska EA, Alvarez-Saavedra E, Townsend M, Yoshii A, Šestan N, Rakic P, Constantine-Paton M, Horvitz HR (2004). Microarray analysis of microRNA expression in the developing mammalian brain. Genome Biol.

[CR114] Ohnuma S-I, Hopper S, Wang KC, Philpott A, Harris WA (2002). Co-ordinating retinal histogenesis: early cell cycle exit enhances early cell fate determination in the Xenopus retina. Development.

[CR115] Olsen PH, Ambros V (1999). The lin-4 regulatory RNA controls developmental timing in Caenorhabditis elegans by blocking LIN-14 protein synthesis after the initiation of translation. Dev Biol.

[CR116] Ono Y, Nakatani T, Sakamoto Y, Mizuhara E, Minaki Y, Kumai M, Hamaguchi A, Nishimura M, Inoue Y, Hayashi H, Takahashi J, Imai T (2007). Differences in neurogenic potential in floor plate cells along an anteroposterior location: midbrain dopaminergic neurons originate from mesencephalic floor plate cells. Development.

[CR117] Otaegi G, Pollock A, Hong J, Sun T (2011). MicroRNA miR-9 modifies motor neuron columns by a tuning regulation of FoxP1 levels in developing spinal cords. J Neurosci.

[CR118] Otaegi G, Pollock A, Sun T (2012). An optimized sponge for microRNA miR-9 affects spinal motor neuron development in vivo. Front Neurosci.

[CR119] Otto SJ, McCorkle SR, Hover J, Conaco C, Han J-J, Impey S, Yochum GS, Dunn JJ, Goodman RHR, Mandel GG (2007). A new binding motif for the transcriptional repressor REST uncovers large gene networks devoted to neuronal functions. J Neurosci.

[CR120] Packer AN, Xing Y, Harper SQ, Jones L, Davidson BL (2008). The bifunctional microRNA miR-9/miR-9* regulates REST and CoREST and is downregulated in Huntington’s disease. J Neurosci.

[CR121] Pang ZP, Yang N, Vierbuchen T, Ostermeier A, Fuentes DR, Yang TQ, Citri A, Sebastiano V, Marro S, Südhof TC, Wernig M (2011). Induction of human neuronal cells by defined transcription factors. Nature.

[CR122] Pang X, Hogan EM, Casserly A, Gao G, Gardner PD, Tapper AR (2014). Dicer expression is essential for adult midbrain dopaminergic neuron maintenance and survival. Mol Cell Neurosci.

[CR123] Peláez N, Carthew RW (2012) Biological robustness and the role of microRNAs. Curr Top Dev Biol 99:237–255 10.1016/B978-0-12-387038-4.00009-4 PMC3746555 22365741

[CR124] Peljto M, Wichterle H (2011). Programming embryonic stem cells to neuronal subtypes. Curr Opin Neurobiol.

[CR125] Petros TJ, Tyson JA, Anderson SA (2011). Pluripotent stem cells for the study of CNS development. Front Mol Neurosci.

[CR126] Philippidou P, Dasen JS (2013). Hox genes: choreographers in neural development, architects of circuit organization. Neuron.

[CR127] Piskounova E, Polytarchou C, Thornton JE, LaPierre RJ, Pothoulakis C, Hagan JP, Iliopoulos D, Gregory RI (2011). Lin28A and Lin28B inhibit let-7 microRNA biogenesis by distinct mechanisms. Cell.

[CR128] Pitto L, Cremisi F (2010). Timing neurogenesis by cell cycle?. Cell Cycle.

[CR129] Rago L, Beattie R, Taylor V, Winter J (2014). miR379-410 cluster miRNAs regulate neurogenesis and neuronal migration by fine-tuning N-cadherin. EMBO J.

[CR130] Reinhardt P, Glatza M, Hemmer K, Tsytsyura Y, Thiel CS, Höing S, Moritz S, Parga JA, Wagner L, Bruder JM, Wu G, Schmid B, Röpke A, Klingauf J, Schwamborn JC, Gasser T, Schöler HR, Sterneckert J (2013). Derivation and expansion using only small molecules of human neural progenitors for neurodegenerative disease modeling. PLoS ONE.

[CR131] Roese-Koerner B, Stappert L, Koch P, Brüstle O, Borghese L (2013). Pluripotent stem cell-derived somatic stem cells as tool to study the role of microRNAs in early human neural development. Curr Mol Med.

[CR132] Rosa A, Brivanlou AH (2011). A regulatory circuitry comprised of miR-302 and the transcription factors OCT4 and NR2F2 regulates human embryonic stem cell differentiation. EMBO J.

[CR133] Rosa A, Spagnoli FM, Brivanlou AH (2009). The miR-430/427/302 family controls mesendodermal fate specification via species-specific target selection. Dev Cell.

[CR134] Rousso DL, Gaber ZB, Wellik D, Morrisey EE, Novitch BG (2008). Coordinated actions of the forkhead protein Foxp1 and Hox proteins in the columnar organization of spinal motor neurons. Neuron.

[CR135] Roy A, Francius C, Rousso DL, Seuntjens E, Debruyn J, Luxenhofer G, Huber AB, Huylebroeck D, Novitch BG, Clotman F (2012). Onecut transcription factors act upstream of Isl1 to regulate spinal motoneuron diversification. Development.

[CR136] Rybak A, Fuchs H, Smirnova L, Brandt C, Pohl EE, Nitsch R, Wulczyn FG (2008). A feedback loop comprising lin-28 and let-7 controls pre-let-7 maturation during neural stem-cell commitment. Nat Cell Biol.

[CR137] Sandoe J, Eggan K (2013). Opportunities and challenges of pluripotent stem cell neurodegenerative disease models. Nat Neurosci.

[CR138] Saurat N, Andersson T, Vasistha NA, Molnár Z, Livesey FJ (2013). Dicer is required for neural stem cell multipotency and lineage progression during cerebral cortex development. Neural Dev.

[CR139] Sempere LF, Freemantle S, Pitha-Rowe I, Moss E, Dmitrovsky E, Ambros V (2004). Expression profiling of mammalian microRNAs uncovers a subset of brain-expressed microRNAs with possible roles in murine and human neuronal differentiation. Genome Biol.

[CR140] Shao N-Y, Hu HY, Yan Z, Xu Y, Hu H, Menzel C, Li N, Chen W, Khaitovich P (2010). Comprehensive survey of human brain microRNA by deep sequencing. BMC Genomics.

[CR141] Shibata M, Kurokawa D, Nakao H, Ohmura T, Aizawa S (2008). MicroRNA-9 modulates Cajal-Retzius cell differentiation by suppressing Foxg1 expression in mouse medial pallium. J Neurosci.

[CR142] Shibata M, Nakao H, Kiyonari H, Abe T, Aizawa S (2011). MicroRNA-9 regulates neurogenesis in mouse telencephalon by targeting multiple transcription factors. J Neurosci.

[CR143] Siegel G, Saba R, Schratt G (2011). MicroRNAs in neurons: manifold regulatory roles at the synapse. Curr Opin Genet Dev.

[CR144] Smidt MP, Burbach JPH (2007). How to make a mesodiencephalic dopaminergic neuron. Nat Rev Neurosci.

[CR145] Smith JR, Vallier L, Lupo G, Alexander M, Harris WA, Pedersen RA (2008). Inhibition of Activin/Nodal signaling promotes specification of human embryonic stem cells into neuroectoderm. Dev Biol.

[CR146] Smith B, Treadwell J, Zhang D, Ly D, McKinnell I, Walker PR, Sikorska M (2010). Large-scale expression analysis reveals distinct microRNA profiles at different stages of human neurodevelopment. PLoS ONE.

[CR147] Stappert L, Borghese L, Roese-Koerner B, Weinhold S, Koch P, Terstegge S, Uhrberg M, Wernet P, Brüstle O (2013). MicroRNA-based promotion of human neuronal differentiation and subtype specification. PLoS ONE.

[CR148] Stern CD (2005). Neural induction: old problem, new findings, yet more questions. Development.

[CR149] Sun GQ, Yu RT, Evans RM, Shi Y (2007). Orphan nuclear receptor TLX recruits histone deacetylases to repress transcription and regulate neural stem cell proliferation. Proc Natl Acad Sci U S A.

[CR150] Sun AX, Crabtree GR, Yoo AS (2013). MicroRNAs: regulators of neuronal fate. Curr Opin Cell Biol.

[CR151] Tabar V, Studer L (2014). Pluripotent stem cells in regenerative medicine: challenges and recent progress. Nat Rev Genet.

[CR152] Tailor J, Kittappa R, Leto K, Gates M, Borel M, Paulsen O, Spitzer S, Karadottir RT, Rossi F, Falk A, Smith A (2013). Stem cells expanded from the human embryonic hindbrain stably retain regional specification and high neurogenic potency. J Neurosci.

[CR153] Vierbuchen T, Ostermeier A, Pang ZP, Kokubu Y, Südhof TC, Wernig M (2010). Direct conversion of fibroblasts to functional neurons by defined factors. Nature.

[CR154] Visvanathan J, Lee S, Lee B, Lee JW, Lee SK (2007). The microRNA miR-124 antagonizes the anti-neural REST/SCP1 pathway during embryonic CNS development. Genes Dev.

[CR155] Volpicelli F, Caiazzo M, Greco D, Consales C, Leone L, Perrone-Capano C, D’Amato LC, di Porzio U (2007). Bdnf gene is a downstream target of Nurr1 transcription factor in rat midbrain neurons in vitro. J Neurochem.

[CR156] Wang Y, Medvid R, Melton C, Jaenisch R, Blelloch R (2007). DGCR8 is essential for microRNA biogenesis and silencing of embryonic stem cell self-renewal. Nat Genet.

[CR157] Wienholds E, Kloosterman WP, Miska E, Alvarez-Saavedra E, Berezikov E, de Bruijn E, Horvitz HR, Kauppinen S, Plasterk RH (2005). MicroRNA expression in zebrafish embryonic development. Science.

[CR158] Wu J, Xie X (2006). Comparative sequence analysis reveals an intricate network among REST, CREB and miRNA in mediating neuronal gene expression. Genome Biol.

[CR159] Wu H, Xu J, Pang ZP, Ge W, Kim KJ, Blanchi B, Chen C, Südhof TC, Sun YE (2007). Integrative genomic and functional analyses reveal neuronal subtype differentiation bias in human embryonic stem cell lines. Proc Natl Acad Sci U S A.

[CR160] Wu Y-C, Chen C-H, Mercer A, Sokol NS (2012). let-7-complex microRNAs regulate the temporal identity of drosophila Mushroom Body neurons via chinmo. Dev Cell.

[CR161] Wurst W, Bally-Cuif L (2001). Neural plate patterning: upstream and downstream of the isthmic organizer. Nat Rev Neurosci.

[CR162] Xi J, Liu Y, Liu H, Chen H, Emborg ME, Zhang SC (2012). Specification of midbrain dopamine neurons from primate pluripotent stem cells. Stem Cells.

[CR163] Xu N, Papagiannakopoulos T, Pan G, Thomson JA, Kosik KS (2009). MicroRNA-145 regulates OCT4, SOX2, and KLF4 and represses pluripotency in human embryonic stem cells. Cell.

[CR164] Xue Y, Ouyang K, Huang J, Zhou Y, Ouyang H, Li H, Wang G, Wu Q, Wei C, Bi Y, Jiang L, Cai Z, Sun H, Zhang K, Zhang Y, Chen J, Fu X-D (2013). Direct conversion of fibroblasts to neurons by reprogramming PTB-regulated microRNA circuits. Cell.

[CR165] Yang D, Li T, Wang Y, Tang Y, Cui H, Zhang X, Chen D, Shen N, Le W (2012). miR-132 regulates the differentiation of dopamine neurons by directly targeting Nurr1 expression. J Cell Sci.

[CR166] Ye W, Shimamura K, Rubenstein JL, Hynes MA, Rosenthal A (1998). FGF and Shh signals control dopaminergic and serotonergic cell fate in the anterior neural plate. Cell.

[CR167] Yekta S, Tabin CJ, Bartel DP (2008). MicroRNAs in the Hox network: an apparent link to posterior prevalence. Nat Rev Genet.

[CR168] Yoo AS, Staahl BT, Chen L, Crabtree GR (2009). MicroRNA-mediated switching of chromatin-remodelling complexes in neural development. Nature.

[CR169] Yoo AS, Sun AX, Li L, Shcheglovitov A, Portmann T, Li Y, Lee-Messer C, Dolmetsch RE, Tsien RW, Crabtree GR (2011). MicroRNA-mediated conversion of human fibroblasts to neurons. Nature.

[CR170] Zhao C, Sun G, Li S, Shi Y (2009). A feedback regulatory loop involving microRNA-9 and nuclear receptor TLX in neural stem cell fate determination. Nat Struct Mol Biol.

[CR171] Zhao C, Sun G, Li S, Lang M-F, Yang S, Li W, Shi Y (2010). MicroRNA let-7b regulates neural stem cell proliferation and differentiation by targeting nuclear receptor TLX signaling. Proc Natl Acad Sci U S A.

[CR172] Zhao C, Sun G, Ye P, Li S, Shi Y (2013). MicroRNA let-7d regulates the TLX/microRNA-9 cascade to control neural cell fate and neurogenesis. Sci Rep.

[CR173] Zheng K, Li H, Zhu Y, Zhu Q, Qiu M (2010). MicroRNAs are essential for the developmental switch from neurogenesis to gliogenesis in the developing spinal cord. J Neurosci.

[CR174] Zheng K, Li H, Huang H, Qiu M (2012). MicroRNAs and glial cell development. Neuroscientist.

